# Wearable-derived heart rate variability and sleep monitoring as predictors of mood episodes in bipolar disorder: a case report

**DOI:** 10.3389/fpsyt.2025.1695158

**Published:** 2025-12-03

**Authors:** Aiko Eto, Keita Mochizuki, Toshikazu Fukami, Wataru Sakakibara, Keisuke Izumi

**Affiliations:** 1 Data-enabled Healthcare Support Center, TechDoctor Inc., Tokyo, Japan; 2 Research Center for Child Mental Development, Chiba University, Chiba, Japan; 3 Sakakibara Occupational Health Partners Co., Ltd., Tokyo, Japan; 4 Division of Rheumatology, Department of Internal Medicine, Keio University School of Medicine, Tokyo, Japan; 5 Division of Rheumatology, Department of Medicine, NHO Tokyo Medical Center, Tokyo, Japan

**Keywords:** bipolar disorder, heart rate variability, digital biomarker, wearable devices, mood episodes, sleep monitoring, case study, depression

## Abstract

**Background:**

Bipolar disorder is a chronic psychiatric condition characterized by alternating episodes of mania and depression, and the prediction and management of mood episodes remain significant clinical challenges. Traditional assessments of mood states have largely relied on subjective methods, such as clinical interviews and self-report questionnaires, which present limitations in terms of early detection and timely intervention. Recently, physiological and behavioral data obtained from wearable devices—particularly heart rate variability (HRV) and sleep parameters—have been proposed as potential digital biomarkers, offering novel opportunities for objective clinical evaluation.

**Case presentation:**

We conducted a single-case study involving a man in his 40s diagnosed with bipolar disorder, who continuously recorded HRV and sleep parameters using a wearable device over approximately eight months. These data were analyzed in relation to self-reported mood scores. The findings revealed that reductions in nocturnal RMSSD preceded the onset of depressive symptoms, while decreases in time spent in bed were significantly associated with the exacerbation of manic symptoms. In contrast, no clear associations were observed between daytime HRV or activity measures and mood scores.

**Conclusion:**

This case study suggests that continuous monitoring of objective physiological measures, such as HRV and sleep parameters, may serve as useful digital biomarkers for predicting mood episodes and preventing relapse in bipolar disorder. Future research involving larger samples and the development of predictive models will be essential to advance the clinical application of these novel assessment approaches.

## 1 Introduction

Bipolar disorder (BD) is a chronic psychiatric disorder with a high recurrence rate, characterized by repeated episodes of mania (or hypomania) and depression (1). Patients may experience periods of remission in which mood symptoms stabilize; however, many continue to suffer from long-term impairments in social and occupational functioning, leading to a reduced quality of life (QoL) (2).

A central challenge in the clinical management of bipolar disorder lies in the control of mood episodes (3). However, the onset of mood episodes is often abrupt, and it is not uncommon for patients to have impaired insight, making it difficult for them to recognize mood changes as pathological (4). Moreover, because mood episodes can be triggered by a variety of factors, including psychosocial stressors and disturbances in sleep–wake rhythms, their clinical management remains highly challenging (5, 6).

In current clinical practice, the assessment of mood states in bipolar disorder relies primarily on psychiatric interviews and patient self-report questionnaires (7). However, these subjective assessments have inherent limitations, as they are influenced by the accuracy of patients’ recollection (recall bias) and distortions in self-evaluation associated with their current mood state (8, 9). Consequently, the lack of objectivity in such assessments poses a risk of delayed detection of mood episodes and hampers timely preventive interventions.

To address these challenges, increasing attention has been directed in recent years toward the application of objective physiological markers in the evaluation of psychiatric disorders. In particular, data continuously collected in daily life through smartphones and wearable devices (WDs) can be used as “digital biomarkers” and are expected to serve as promising novel tools for clinical assessment (10). Among these, heart rate variability (HRV) serves as a non-invasive measure of autonomic nervous system regulation of cardiac function.

HRV is derived from the analysis of fluctuations in the intervals between successive heartbeats (RR intervals) and is commonly categorized into three domains: time-domain, frequency-domain, and non-linear measures. Within the time-domain indices, the standard deviation of normal-to-normal intervals (SDNN) and the root mean square of successive differences (RMSSD) are representative measures, with RMSSD in particular regarded as a marker of parasympathetic activity (11, 12).

Associations between HRV and psychiatric disorders, such as depression and anxiety disorders, have been reported. For example, patients with depression tend to exhibit reduced HRV, with particularly pronounced decreases in RMSSD and the HF component, both of which reflect parasympathetic activity (13). Similarly, reduced HRV has also been observed in anxiety disorders, and it has been suggested that excessive sympathetic predominance may contribute to their pathophysiology (14, 15). These findings suggest that an imbalance in the autonomic nervous system constitutes a physiological basis for mood disorders and anxiety symptoms.

In bipolar disorder, an increasing body of research suggests an association between HRV and mood states. For example, it has been reported that patients with bipolar disorder exhibit reduced HRV compared to healthy controls, even during remission (16). Furthermore, regarding the association with mood episodes, multiple studies have consistently demonstrated that HRV is significantly reduced during depressive states (13, 17). In contrast, although findings on HRV alterations during manic episodes have been inconsistent, it has been suggested that manic states may exhibit autonomic activity patterns distinct from those observed in depressive states (18). These studies suggest that HRV may reflect the pathophysiology of bipolar disorder and could serve as a biomarker for the objective assessment of mood states.

In recent years, the widespread use of commercial wearable devices, such as Fitbit and Oura Ring, has facilitated the collection of high-frequency and long-term HRV data in daily life. This advancement has enabled longitudinal analyses of HRV in large cohorts, which were previously challenging in conventional clinical studies, thereby contributing to a better understanding of the pathophysiology of psychiatric disorders, including bipolar disorder, and opening possibilities for real-time monitoring of mood states (19, 20). In the future, HRV analysis using wearable devices is expected to play an important role as an objective assessment tool in clinical practice, with the potential to support personalized medicine and preventive interventions.

The aim of this study is to continuously collect HRV data using a wearable device and to explore its association with fluctuations in subjective depressive symptom scores in a single case of a patient with bipolar disorder. Through this research, we seek to examine whether HRV monitoring in daily life could serve as an objective indicator of mood fluctuations and, consequently, be applicable as a potential digital biomarker for relapse prevention. This case-level approach provides a complementary perspective to large-cohort studies by capturing individual-level dynamics in detail. Furthermore, such a case-level approach can help identify which sensing modalities are most promising for future large-scale proof-of-concept studies.

## 2 Case presentation

The participant was a man in his 40s diagnosed with bipolar disorder. The purpose and procedures of the study were thoroughly explained, and written informed consent was obtained. He had first been diagnosed with bipolar disorder by another physician 15 years earlier, and under the care of his current psychiatrist meets the DSM-5 diagnostic criteria for bipolar disorder. At the time of his initial diagnosis, he was treated at a university hospital, where his primary psychiatrist changed every few months. Subsequently, he transferred to his current clinic and has since been under the continuous care of the same psychiatrist for more than 10 years. He attends consultations lasting approximately 10–15 minutes each.

The patient was prescribed multiple psychiatric medications, including mood stabilizers, anxiolytics, antidepressants, antipsychotics, and sleep medications. A detailed list of prescribed medications, including dosage and class, is provided in **Supplementary Table 1**. Medication adherence was generally good, supported by family assistance, with occasional brief lapses during periods of disrupted daily routines, sometimes followed by transient symptom exacerbations.

In recent years, the patient has experienced approximately three to four episodes annually, predominantly occurring between spring and early summer, characterized by hypomanic to manic symptoms. Each episode typically lasts one to two weeks, and the patient is aware that his cycles are relatively rapid, although he has not met the diagnostic threshold for rapid cycling. Recurrences of manic episodes are commonly preceded by disturbances in sleep–wake rhythm (particularly delayed sleep onset and waking), fluctuations in activity levels and performance (tending toward decline), heightened emotional reactivity within the household (including irritability and raising his voice), and impulsive behaviors such as unplanned shopping or going out. While he has managed to maintain adequate functioning in social settings outside the home, impulsivity and aggression have been particularly prominent in family interactions. With regard to depressive episodes, the patient’s self-reports and records provide less detailed information on frequency and duration compared with hypomanic or manic states; however, several characteristic features have been noted. He has experienced multiple periods of depression in the past, during which both family feedback and daily life records documented intermittent symptoms such as low mood, lack of energy, and diminished motivation. During depressive states, transient declines in sleep quality were observed, along with relative reductions in daytime activity levels (e.g., step counts and frequency of going out). Reports have also indicated decreased work efficiency, difficulties in self-care and household tasks, and reduced engagement with family members. Notably, depressive episodes were sometimes temporally associated with stressful family circumstances.

## 3 Methods

The participant was instructed to wear a wearable device (Fitbit Charge 6, Google LLC, Mountain View, CA, USA) on the wrist opposite to the dominant hand for as many hours as possible throughout the day, enabling continuous recording of activity levels, sleep patterns, and heart rate under naturalistic conditions. The collected data were first transmitted to the participant’s smartphone and subsequently uploaded to the manufacturer’s cloud server via an internet connection. The data were then transferred to the cloud-based analysis platform SelfBase™ (TechDoctor Inc., Tokyo, Japan). Within SelfBase, the data underwent anonymization and preprocessing procedures, including data cleaning, and were then formatted into a dataset suitable for research purposes. The monitoring period spanned approximately eight months, from February to October 2024.

For subjective mood assessment, the participant used the mood-tracking application eMoods to rate four domains: depressed mood (DM), elevated mood (EM), irritability, and anxiety. Each domain was self-rated on a 4-point Likert scale ranging from 1 (none) to 4 (severe), with 2 (mild) and 3 (moderate) as intermediate levels. In this study, the DM and EM scores were used as the primary variables for analysis, while the other scores were employed as supplementary measures (21).

For the calculation of HRV, photoplethysmography (PPG)-derived heart rate data obtained at several-second intervals were used to compute RR intervals, which were then analyzed separately for sleep and daytime periods. HRV was aggregated in 15-minute windows, with RMSSD adopted as the primary index. RMSSD represents the square root of the mean squared differences of successive RR intervals and is considered an indicator of parasympathetic activity. In addition, SDNN was calculated in an exploratory manner to examine its relationship with mood scores and its day-to-day variability. For daily RMSSD values, the mean and standard deviation across the entire observation period were computed. Days with HRV values exceeding ±1.5 standard deviations from the individual mean were defined as abnormal, following a previous HRV study (22).

To examine the relationship between HRV and mood scores, as a prospective evaluation, days with abnormal HRV were defined as index days (day 0), and the proportion of days within 7 days including the day on which any mood score (DM/EM/Irritability/Anxiety) was recorded as ≥2 was calculated. In this case, if mood records were missing (value of 0) and no observations were available during the 7-day period, the index day was excluded from the denominator. As a retrospective evaluation, days on which any mood score was recorded as ≥2 were defined as index days (day 0), and the proportion of days within the preceding 7 days including the day on which abnormal HRV occurred was calculated. In both evaluations, the denominator (observable days) was defined as the days on which mood records were actually observed, and the primary indicator was the proportion (%) of such days.

Sleep data were obtained using the automatic sleep detection algorithm of the wearable device, with the estimated interval from bedtime to wake-up time defined as time in bed (TIB). In this study, only the main sleep was analyzed, and naps were excluded. Sleep stages (wake, REM, light, deep) and sleep onset and offset times were based on estimates provided by the Fitbit algorithm. For sleep evaluation, the relationship between mood scores and TIB was examined. TIB was calculated on a daily basis, and a time series smoothed using a 4-week moving average was constructed. The smoothed TIB values were then compared with mood scores to examine variation trends over the observation period. Furthermore, as a statistical analysis, the relationship between TIB and mood scores (DM, EM) was examined using group comparisons. Specifically, participants were divided into two groups, DM = 1 *vs*. DM ≥ 2 and EM = 1 *vs*. EM ≥ 2, and the mean TIB between groups was compared using Welch’s t-test (equal_var = False). Effect sizes were calculated as Cohen’s d and Hedges’ g (with small-sample correction), and 95% confidence intervals for Hedges’ g were estimated using the bootstrap method with 10,000 iterations. In addition, to account for potential seasonal effects, linear regression analysis (Ordinary Least Squares; OLS) including month as a categorical covariate was performed to examine the independent association between EM and TIB. A two-tailed p-value of < 0.05 was considered statistically significant.

The analyses were conducted in an exploratory manner, aligning HRV indices, sleep parameters, and mood scores on a daily basis, and examining variation patterns using descriptive statistics and time-series plots. The study did not aim to test causal relationships; rather, interpretations were based on relative intra-individual changes. Days when the participant did not wear the device or when sufficient sleep data were not available according to the Fitbit algorithm were treated as missing and excluded without imputation.

All analyses and visualizations were performed using Python (version 3.12), primarily employing pandas (v2.2.2), numpy (v1.26.0), and plotly (v5.24.1). For statistical analyses, scipy (v1.16.1) and statsmodels (v0.14.5) were used.

## 4 Results

**Figure 1:**
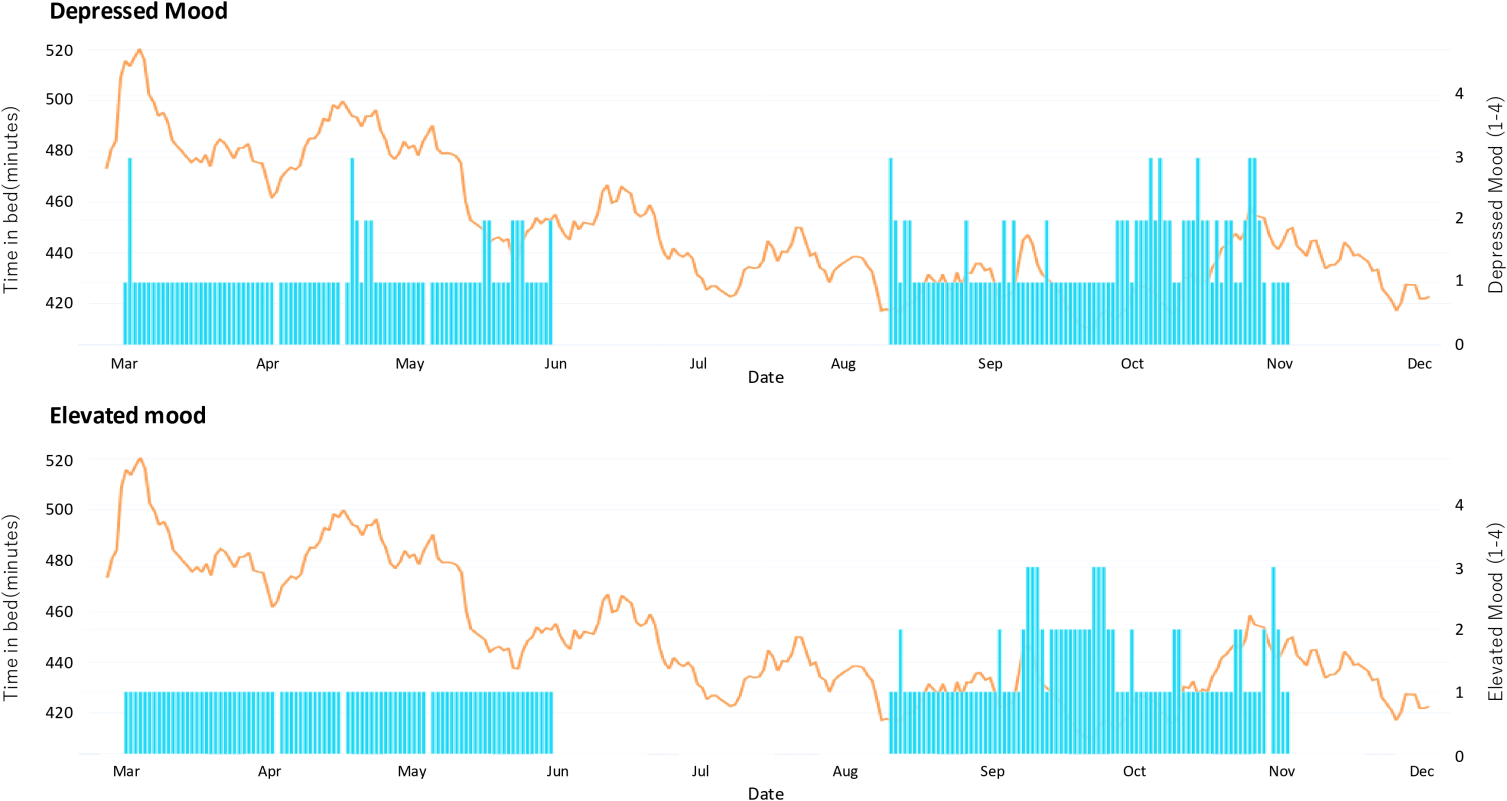
Time in bed and daily mood ratings.

Mood scores were rated on a 4-point scale ranging from 1 (none) to 4 (severe). **Figure 1** illustrates the trajectories of DM scores, EM scores, and the 4-week moving average of TIB across the observation period. No significant association was observed between DM scores and TIB. In contrast, EM scores tended to increase in parallel with reductions in sleep duration, and a significant difference in TIB was found between the EM = 1 and EM ≥ 2 groups (Welch’s t-test, mean difference = 60.6 minutes, 95% CI [31.7, 89.6]; Hedges’ g = 0.80). Furthermore, in the OLS regression analysis adjusted for monthly seasonality as a covariate, the effect of EM remained independently significant (β = -56.2, 95% CI [-98.9, -13.4], p = 0.010, R² = 0.107), indicating that higher EM was associated with shorter TIB even after accounting for seasonal factors.

**Figure 2:**
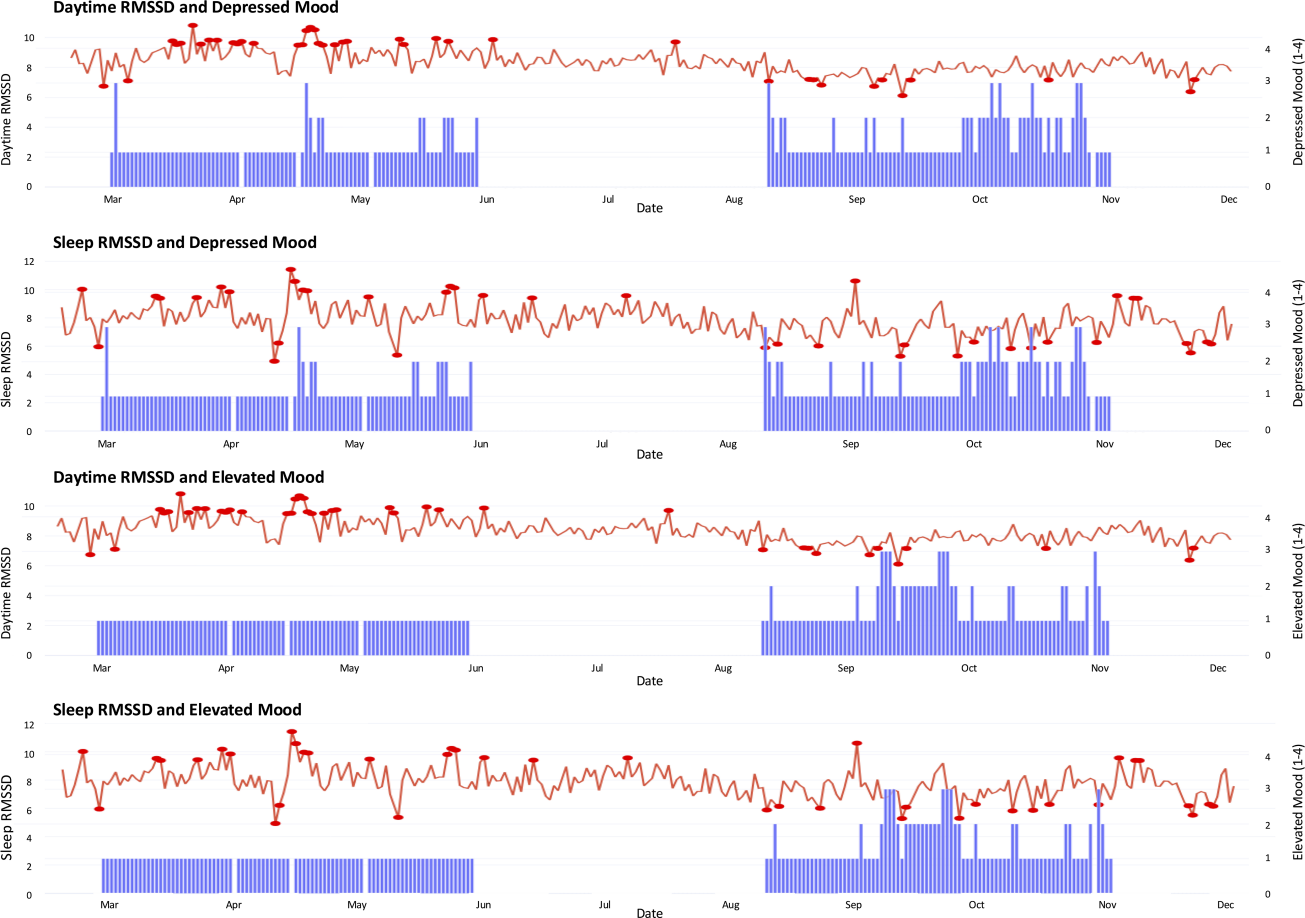
Daytime and sleep RMSSD with daily mood ratings.

**Figure 2** shows the trajectories of DM and EM scores along with nocturnal RMSSD and daytime RMSSD. During the observation period, the participant experienced multiple episodes of elevated DM scores (≥2). A temporal association was observed between decreases in nocturnal RMSSD and increases in DM scores. Specifically, there were 14 days on which RMSSD declined by more than 1.5 standard deviations below the individual baseline, and on 13 of those days (86.67%), an increase in DM scores (≥2) was recorded within the subsequent 7 days.

Furthermore, among the 43 days on which a DM score ≥ 2 was recorded, 32 days (74.42%) were preceded within 7 days by a decline in RMSSD of more than 1.5 standard deviations below the individual baseline (**Table 1**). SDNN showed a similar trend to RMSSD, although RMSSD demonstrated a stronger association (**Supplementary Figure 1**). In contrast, fluctuations in daytime RMSSD were not clearly associated with DM scores. Activity measures (e.g., step count, calorie expenditure) also showed no consistent relationship with mood scores.

**Table 1:** Abnormal HRV days and mood worsening counts.

HRV metric	Number of abnormal days (entire observation period)	Number of abnormal days (with mood ratings available)	Number of days with mood worsening within 1 week after abnormality	Percentage of days with mood worsening within 1 week after abnormality	Number of days with mood worsening	Number of abnormal days in the 1-week period prior to mood worsening	Percentage of abnormal days in the 1-week period prior to mood worsening
CVRR	16	13	10	76.92	43	22	51.16
SDNN	17	14	12	85.71	43	24	55.81
RMSSD	19	15	13	86.67	43	32	74.42
CVI	20	15	13	86.67	43	28	65.12
CSI	20	13	10	76.92	43	28	65.12
SD1	19	15	13	86.67	43	32	74.42
SD2	16	13	11	84.62	43	24	55.81
avgHR	7	4	3	75.00	43	5	11.63
diffHR	17	13	7	53.85	43	19	44.19

CVRR, Coefficient of Variation of RR intervals; SDNN, Standard Deviation of NN intervals; RMSSD, Root Mean Square of Successive Differences; CVI, Cardiac Vagal Index; CSI, Cardiac Sympathetic Index; SD1, Standard Deviation 1 (short-term HRV; derived from Poincaré plot); SD2, Standard Deviation 2 (long-term HRV; derived from Poincaré plot); avgHR, Average Heart Rate; diffHR, difference between maximum and minimum heart rate within a defined period.

## 5 Discussion

In this case study, HRV and sleep data were continuously collected in daily life using a commercial wearable device in combination with a mood-tracking application, and their associations with subjective mood scores were examined in an exploratory manner. The findings demonstrated two key points: (i) decreases in nocturnal RMSSD tended to precede the worsening of depressive symptoms; (ii) shorter sleep duration was associated with increased severity of elevated mood symptoms. In contrast, no clear associations were observed between daytime HRV or activity measures and mood scores.

A significant association was observed between reduced TIB and increased EM scores. Consistent results were obtained in both group comparisons and OLS regression analyses, and this association remained independent even after adjusting for seasonality. Previous studies have reported that shortened sleep duration and disrupted sleep–wake rhythms can trigger manic or hypomanic episodes in bipolar disorder, and the observation of a similar tendency in the present case is clinically meaningful. These findings suggest that sleep monitoring may contribute to the early detection and prevention of recurrence of mood elevation.

Previous studies have demonstrated an association between reduced HRV and depressive symptoms (23, 24), and in the present study, decreases in nocturnal RMSSD were shown to precede the onset of depressive symptoms. Although no clear relationship was observed for daytime RMSSD, this may be attributable to its susceptibility to external noise such as from activity levels and psychosocial factors. Nocturnal HRV, particularly RMSSD provides a more stable assessment of parasympathetic activity, and thus may serve as a promising digital biomarker for anticipating depressive episodes.

The findings of this case study indicate that HRV and sleep monitoring using wearable devices may contribute to the prediction of mood episodes in bipolar disorder. In particular, the observation that decreases in nocturnal RMSSD preceded the worsening of depressive symptoms by several days, and that reductions in TIB were associated with heightened mood elevation, suggests that distinct physiological precursors may exist for different types of mood episodes. Traditionally, the clinical assessment of bipolar disorder has relied on subjective self-reports; however, the integration of objective data derived from wearable devices holds the potential to be implemented in clinical practice as an early warning system and as a trigger for preventive interventions.

Recent large-scale studies have demonstrated that mood states and relapse risk in bipolar disorder can be predicted from physiological signals obtained via wearable devices, using complex data analytic techniques including machine learning (**Table 2**). While these studies highlight the promise of data-driven prediction at the cohort level, the present single-case analysis provides a complementary perspective by elucidating detailed within-individual temporal dynamics that may be overlooked in large-scale studies.

**Table 2:** Summary of recent wearable-derived physiological studies in bipolar disorder.

Study	Sample size and diagnoses	Follow-up duration	Devices	Passive data	Subjective data	Outcome
Ortiz et al. (2025) (25)	N=50 (BD I:31, BD II:19)	median 495.0 days (IQR:410.0)	Oura ring Generation 2	activity, sleep	ASRM	Early indicators of hypomanic episode onset were within-night variability in sleep stages and within-day variability in activity levels. Among the variability metrics, the 12-hour fluctuations in sleep stages provided the earliest warning, achieving an accuracy of 0.87; this was followed by 12-hour fluctuations in activity levels, with an accuracy of 0.89. On a day-to-day basis, sleep variability predicted hypomanic onset a median of 3.0 days before, while activity variability predicted onset a median of 2.5 days beforehand.
Ortiz et al. (2025) (26)	N=127 (BD I:83, BD II:44)	383.5 days (SD:175.3)	Oura ring Generation 2	activity, sleep	PHQ-9	Day-to-day variability in the number of steps was the earliest indicator of the onset of depressive symptoms. Early Detection Days: activity variability: median −7.0 days (IQR 9.0); sleep variability (deep sleep): median −4.0 days (IQR 5.0).
Wu et al. (2025) (27)	N=24 (BD)	6.39 months (SD:4.85)	Garmin Vivosmart 4	sleep, activity, HR	BDI, YMRS	The XGBoost-based model for predicting depressive symptoms showed an accuracy of 0.83 when individual-specific characteristics were integrated. The manic symptom prediction model exhibited an accuracy of 0.91 with the inclusion of personalized parameters. SHAP-based feature importance analysis revealed that increased resting HR, decreased physical activity (fewer than 6000 steps daily), and short sleep duration (under 6 hours) were the most influential factors in detecting depression.
Pascual et al. (2025) (28)	N=139 (BD:104, HC:35)	48 hours, 1-2times	Empatica E4	ACC, TEMP	YMRS, HDRS, SOFAS, PANSS	TEMP was consistently higher in patients with ME than in the rest of the groups during waking hours. In the depressed group, TEMP did not differ significantly between the acute and remission phases, and likewise showed no significant difference from healthy controls.
Lipschitz et al. (2025) (29)	N=54, (BD I:41,BD II:13)	9months	Fitbit Inspire	sleep, activity, HR	ASRM, PHQ-8	Using the BiMM Forest approach, the model achieved an ROC-AUC of 86.0 percent for detecting depressive episodes and 85.2 percent for identifying hypomanic episodes.
Song et al. (2024) (30)	N=139 (MDD:45, BD I:35, BD II:59)	290 days (SD:297)	Fitbit Charge HR, 2 or 3,	sleep	Daily self-reports of mood via smartphone-based EMA, rating depressive and elevated mood	Among patients with MDD and BD I, evidence of circadian phase influencing mood was identified in 66.7% of MDD cases and 85.7% of BD-I cases. In contrast, the reverse direction—mood affecting circadian phase—was rarely observed. For those with BD II, the proportion showing circadian-to-mood causal influence remained under 37%, with no consistent pattern emerging across patients.
Lim et al. (2024) (6)	N=168 (MDD: 57, BDI:42, BDII:69)	587 days (SD:374)	Fitbit Charge HR, 2 or 3,	sleep	MADRS,YMRS, CSM, GSS	ML-based accurate next-day prediction of mood episodes. The XGBoost classifier demonstrated strong discriminative ability, achieving area under the ROC curve values of 0.925 for predicting depressive episodes, 0.984 for manic episodes, and 0.985 for hypomanic episodes.
Anmella et al. (2024) (31)	N=68 (BD:49, HC:19)	48 hours, 1-2times	Empatica E4	EDA	YMRS, HDRS	In the depressive cohort, mean electrodermal activity was significantly lower than in the manic, remission, and healthy control groups (p = 0.003). In the manic cohort, mean EDA decreased significantly following remission (p = 0.035), suggesting a reduction in sympathetic nervous system arousal as symptoms improved.
Corponi et al. (2024) (24)	N=23 (BD)	48 hours, 3-4times	Empatica E4	ACC, BVP, EDA, HR, IBI, TEMP	YMRS, HDRS	The natural logarithm of the nocturnal average RMSSD (lnRMSSD) was suggested to quantitatively capture autonomic function recovery in patients with bipolar disorder and may serve as an adjunct for assessing treatment response and relapse risk.
Zakariah et al. (2023) (32)	N=55 (MDD+BD I+ BD II:23, HC:32)	13 days	Actiwatch	activity	MADRS	A model combining UMAP and a neural network classified MDD, BD, and HCs with 99.1% accuracy (F1 = 0.99, κ = 0.98).
Zhang et al. (2023) (33)	N=30 (BD:30)	3–10 days	ActiGraph wGT3X-BT	activity	YMRS, HDRS	The mean activity levels during the relatively long monitoring period and the intra-day variation within the groups could reflect the differences in motor activity. The average activity level over 24 hours was greatest in the manic group and was significantly higher than in both the depressive and mixed-state groups.
Lee et al. (2023) (34)	N=270 (MDD:95, BD I:78, BD II:97)	279.7 days (SD:263.5)	Fitbit Charge HR, 2 or 3, smartphone	sleep, activity, HR, Light exposure	Psychiatrist’s clinical assessment (Daily self-reports of mood via smartphone-based EMA)	The model anticipated the emergence of major depressive, manic, and hypomanic episodes within a three-day window with accuracies exceeding 90%.

MDD, Major Depressive Disorder; BD I, Bipolar I Disorder; BD II, Bipolar II Disorder; HC, Healthy Controls; PHQ-8, Patient Health Questionnaire-8; HDRS, Hamilton Depression Rating Scale; MADRS, Montgomery-Asberg Depression Rating Scale; ASRM, Altman Self-Rating Mania Scale; YMRS, Young Mania Rating Scale; CSM, Composite Scale of Morningness; GSS, Global Seasonality Score; EMA, Ecological Momentary Assessment; EDA, Electrodermal activity; IBI, Inter-Beat Intervals; ACC, 3D Acceleration; BVP, Blood Volume Pressure; HR, Heart Rate; lnRMSSD:natural logarithm of the root mean square of successive differences of RR intervals; TEMP, Skin Temperature; UMAP, Uniform Manifold Approximation and Projection; IQR, Interquartile Range.

This study is notable for its novelty and significance in that it combined a commercial wearable device with a smartphone application to continuously collect data over an extended period of eight months and analyzed intra-individual changes in relation to mood scores. In particular, there are limited prior reports in bipolar disorder that have demonstrated physiological markers associated with both depressive and elevated mood symptoms within the same individual case.

This study was an exploratory analysis based on a single case, and thus its generalizability is limited. In addition, mood scores relied on self-reporting, which is inevitably subject to recall bias and fluctuations in self-assessment. Moreover, HRV indices were calculated using PPG-derived heart rate data obtained from Fitbit. However, the details of Fitbit’s signal processing procedures (e.g., preprocessing, artifact removal) leading to heart rate estimation are not disclosed, and upstream estimation errors may have influenced the HRV indices. Furthermore, potential confounding factors, such as medication adherence and lifestyle habits, could not be fully controlled, and these may have influenced both physiological and mood measures. Additionally, the monitoring period was limited to approximately eight months (February–October 2024) because the available data set covered only this time frame. As a result, data from the winter season, when depressive episodes are more likely to occur, were not captured, which limits the ability to evaluate potential seasonal effects on mood episodes and HRV. As this study utilized data collected and analyzed on an industry-affiliated platform, independent replication by external groups will be essential to ensure reliability and generalizability.

Further research should involve prospective studies with larger samples to quantitatively examine the associations between HRV, sleep parameters, and mood episodes. Future studies with year-long or multi-year monitoring across different seasons are warranted to confirm the influence of seasonal patterns in bipolar disorder. Controlling for potential confounders will also be important. In addition, the development of multivariate predictive models using machine learning, as well as comparative validation with medical-grade devices, will be necessary to enhance clinical applicability. Finally, advancing research aimed at identifying distinct physiological precursors for different types of mood episodes may contribute to the personalization of care in bipolar disorder.

## Data Availability

The original contributions presented in the study are included in the article/**Supplementary Material**. Further inquiries can be directed to the corresponding author.

## Supplementary material

Supplementary Table 1 Medication details for the case participant.
